# Efficacy Profiles of Antimicrobials Evaluated against *Staphylococcus* Species Isolated from Canine Clinical Specimens

**DOI:** 10.3390/ani11113232

**Published:** 2021-11-12

**Authors:** Daniel Nenene Qekwana, Agricola Odoi, James Wabwire Oguttu

**Affiliations:** 1 Section of Veterinary Public Health, Department of Paraclinical Sciences, Faculty of Veterinary Science, University of Pretoria, Pretoria 0110, South Africa; 2 Biomedical and Diagnostic Sciences, College of Veterinary Medicine, University of Tennessee, Knoxville, TN 37996, USA; aodoi@utk.edu; 3 Department of Agriculture and Animal Health, College of Agriculture and Environmental Sciences, University of South Africa, Johannesburg 709, South Africa; Joguttu@unisa.ac.za

**Keywords:** antimicrobial resistance, efficacy, *Staphylococcus*, principal components analysis, PCA, factor analysis, eigenvalues

## Abstract

**Simple Summary:**

Clinical cases associated with staphylococci infections are common among dogs and cats. There is evidence to suggest that staphylococci infections are increasingly becoming unresponsive to commonly used antimicrobials. This negatively impacts the ability of these infections to be treated successfully. Although resistance among these organisms has been linked to several factors, including sharing the same mechanism of action or belonging to the same group, there is evidence to suggest that cross resistance can occur between unrelated antimicrobials. The findings of this study not only confirm that antimicrobials that belong to the same group share the same mechanism of resistance and similar antimicrobial efficacy against staphylococcal infections, but also show that cross resistance occurs between unrelated antimicrobials. This should be taken into consideration when selecting antimicrobials for inclusion in the susceptibility testing panel as well as for the treatment of staphylococci infections.

**Abstract:**

Cross-resistance occurs between antimicrobials with either similar mechanisms of action and/or similar chemical structures, or even between unrelated antimicrobials. This study employed a multivariate approach to investigate the associations between the efficacy profile of antimicrobials and the clustering of eleven different antimicrobial agents based on their efficacy profile. Records of the susceptibility of 382 confirmed *Staphylococcus* species isolates against 15 antimicrobials based on the disc diffusion method were included in this study. Tetrachoric correlation coefficients were computed to assess the correlations of antimicrobial efficacy profiles against *Staphylococcus aureus*. Principal components analysis and factor analysis were used to assess the clustering of antimicrobial susceptibility profiles. Strong correlations were observed among aminoglycosides, penicillins, fluroquinolones, and lincosamides. Three main factors were extracted, with Factor 1 dominated by the susceptibility profile of enrofloxacin (factor loading (FL) = 0.859), gentamicin (FL = 0.898), tylosin (FL = 0.801), and ampicillin (FL = −0.813). Factor 2, on the other hand, was dominated by the susceptibility profile of clindamycin (FL = 0.927) and lincomycin-spectinomycin (FL = 0.848) and co-trimazole (FL = −0.693). Lastly, Factor 3 was dominated by the susceptibility profile of amoxicillin-clavulanic acid (FL = 0.848) and cephalothin (FL = 0.824). Antimicrobials belonging to the same category or class of antimicrobial, tended to exhibit similar efficacy profiles, therefore, laboratories must choose only one of the antimicrobials in each group to help reduce the cost of antimicrobial susceptibility tests.

## 1. Introduction

Infection with *Staphylococcus* species is common in domestic animals including dogs and cats [1,2]. These organisms cause various clinical conditions that include pyoderma, otitis, and wound infections [3,4,5,6]. However, there are increasing reports of antimicrobial resistance among *Staphylococcus* isolates in veterinary settings [1,7]. These are likely to complicate treatment outcomes due to treatment failures and the resultant poor prognosis, high morbidities, and mortalities [8].

The increasing prevalence of resistance has been attributed to over prescription, improper prescription, and acquisition of resistant genes through a number of mechanisms including plasmids [9,10,11]. There is also evidence of association between the resistance profile of antimicrobials that belong to the same category and also with antimicrobials that belong to other categories [12,13,14]. For example, cross resistance has been reported between members of the β-lactams, fluroquinolones, and aminoglycosides [15]. Cross-resistance has also been reported against antimicrobial drugs to which bacteria have not previously been exposed [16]. This may develop without the target mutations or may develop mediated by mutation in the target resistance protein like in the case of fluoroquinolone. The latter has been associated with resistance among multiple non-quinolone. Research by Pal et al. [17] on cross-resistance among unrelated antimicrobials provides information on the long-term efficacy of novel antimicrobial compounds. In view of this, studies that investigate cross resistance among related and unrelated antimicrobial groups are needed.

Although several studies have investigated associations between the efficacy profiles of antimicrobials in relation to *Staphylococcus* isolates in human medicine, similar studies are lacking in veterinary medicine in South Africa. In addition, there are limited studies that have investigated this phenomenon using statistically rigorous methods such as multivariate techniques. This study investigated the association between the efficacy profiles of antimicrobials evaluated against *Staphylococcus* clinical isolates. Study findings contribute to improved understanding of cross resistance among clinical isolates and can be used to determine antimicrobials for use in veterinary practice, especially in low resource settings.

## 2. Materials and Methods

### 2.1. Data Source

This study used retrospective secondary data of the susceptibility profile of 382 confirmed *Staphylococcus* species isolates from canine clinical cases presented at a veterinary academic hospital located in Pretoria between January 2007 and December 2012. The culture and sensitivity analysis were conducted by the bacteriology laboratory of the veterinary academic hospital. The dataset was assessed for duplicate entries, missing data, and inconsistencies, such as improbable values.

### 2.2. Antimicrobial Susceptibility Testing

All the isolates were subjected to antimicrobial susceptibility testing (AST) against a panel of 15 drugs using the disc diffusion method following the guidelines of the Clinical and Laboratory Standards Institute [18,19,20,21,22,23,24]. The panel included the following antimicrobials: 30 μg amikacin, 30 μg doxycycline, 5 μg enrofloxacin, 10 μg gentamicin, 10 μg ampicillin, 10 μg penicillin G, 25 μg trimethoprim-sulfamethoxazole (co-trimoxazole), 30 μg chloramphenicol, 30 μg cephalothin, 30 μg kanamycin, 2 μg clindamycin, 100 μg lincospectin (LS100), 5 μg orbifloxacin, 20/10 μg amoxicillin/clavulanic acid and 15 μg tylosin. The laboratory that supplied the data classified the susceptibility profile of the isolates into three categories (i.e., susceptible, intermediate, or resistant) in accordance with the Clinical and Laboratory Standards Institutes [18,19,20,21,22,23,24]. However, for the purposes of this study, intermediate and resistance isolates were recoded as nonsusceptible for all subsequent analyses.

### 2.3. Data Analyses

#### 2.3.1. Correlation Analysis

Tetrachoric correlation coefficients were computed to assess the relationship between the susceptibility profiles of different antimicrobials. The tetrachoric correlation coefficients were computed in this study because of their appropriateness for assessment of correlations between dichotomous variables [25] and are indicated in situations where consistency measures of reliability are preferred to agreement measures [26]. In this study, pairs of antimicrobials with correlation coefficients of ≥0.7 were classified as highly correlated. If this was observed between agents belonging to the same antimicrobial category, only one of the two was selected for inclusion in the subsequent principal component analysis (PCA) and factor analysis.

#### 2.3.2. Principal Components and Factor Analyses

Principal components analysis (PROC PRINCOM) and factor analysis (PROC FACTOR), implemented in SAS 9.4 (SAS Institute Inc., Cary, NC, USA), were used to assess the relationship between the efficacy profiles of antimicrobials against *Staphylococcus* species. Eigenvalues >1 were used to determine the number of factors to be retained in the factor analysis. In addition, the scree plot was used to visualize the factor numbers and associated eigenvalues. Orthogonal axis rotation (varimax) was applied to the factors to allow for easy interpretation of the interrelationships between the antimicrobial agents. The reliability of the items was assessed using the McDonald’s omega coefficient test implemented in JASP software version 0.14.1.0 (University of Amsterdam, Amsterdam, the Netherlands) [27]. Variables with low communality values were removed from the PCA.

## 3. Results

High numbers of nonsusceptible isolates were observed for ampicillin (58.9%), penicillin (55.5%), lincospectin (44.5%), and clindamycin (37.43%). However, low numbers of nonsusceptible isolates were observed for aminoglycoside (9.2%), tetracyclines (15.7%), fluoroquinolones (%), potentiated sulfonamides (17.02%), amphenicols (11.34), amoxicillin-clavulanic acid (12.57%) and macrolide (10.47%) (Table 1).

Strong correlations were observed between efficacy profiles of the following antimicrobials: amikacin vs. gentamycin (*r* = 0.79), amikacin vs. kanamycin (*r* = 0.72), kanamycin vs. gentamycin (*r* = 0.93), ampicillin vs. penicillin (*r* = 0.96), enrofloxacin vs. orbifloxacin (*r* = 0.91), and lincospectin vs. clindamycin (*r* = 0.79) (Table 2).

**Figure 1:**
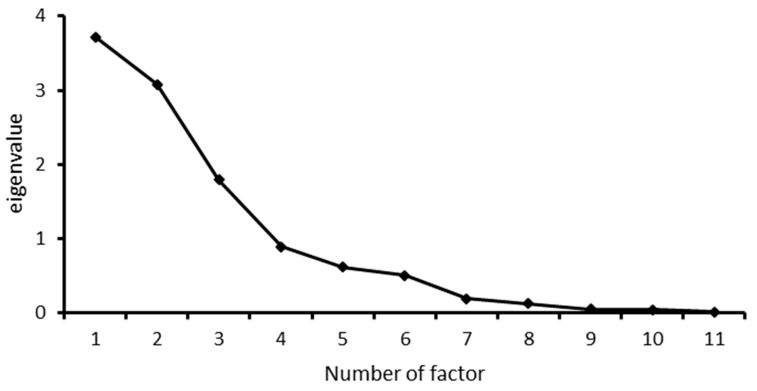
Scree plot showing factors that had eigenvalues >1 from a study of efficacy profiles of antimicrobials evaluated against *Staphylococcus* isolated from canine clinical specimens in South Africa.

Three factors had eigenvalues >1 and were, therefore, extracted (Table 3). These factors together accounted for 85% of variation in antimicrobial nonsusceptibility (Figure 1, Table 3).

Factor 1 was dominated by enrofloxacin (factor loading (FL) = 0.859), gentamicin (FL = 0.898), tylosin (FL = 0.801), and ampicillin (FL = −0.814). Factor 2 was dominated by clindamycin (FL = 0.927), lincomycin-spectinomycin (FL = 0.848) and co-trimazole (FL = −0.693). Lastly, Factor 3 was dominated by amoxicillin-clavulanic acid (FL = 0.848) and cephalothin (FL = 0.824) (Table 4). McDonald’s omega values indicated good internal reliability of the items (Table 5).

## 4. Discussion

This study investigated the interrelationships between the efficacy profiles of antimicrobial agents against *Staphylococcus* isolates. Strong correlations between the efficacy profiles were observed between antimicrobials that belong to the same category. For example, strong correlations were observed between efficacy profiles of members of each of the aminoglycoside, fluoroquinolone, and penicillin categories of antimicrobials. This was expected because antimicrobials that share a similar mechanism of action or chemical structure are known to exhibit cross resistance [11,13,28,29,30]. Therefore, if a member of one of the categories ceases to be efficacious against a pathogen, other members of that category are most unlikely to be efficacious against the same organisms. In view of this, during in vitro testing, antimicrobials belonging to the same group or class of antimicrobials should not be included in the testing panel. Only one antimicrobial from the group should be selected to represent other members of the group sharing a similar chemical structure and/or mechanism of action. This would be a cost-cutting measure with the potential to make antimicrobial sensitivity testing more accessible and affordable, especially in low resource settings. Furthermore, the results of the study suggest that clinicians should not consider antimicrobials for the treatment of *Staphylococcus aureus* if such antimicrobials belong to the same group of antimicrobials against which low efficacy against *Staphylococcus* isolates have been observed. This is likely to result in treatment failure.

Results of factor analysis showed that enrofloxacin, gentamycin, tylosin and ampicillin clustered together, suggesting a similarity in the efficacy profiles of these groups of antimicrobials. However, ampicillin compared to the other antimicrobials tended to load negatively. This suggests that unlike the other groups of antimicrobials, ampicillin had an efficacy profile that was opposite in relation to *Staphylococcus* species. This could be explained by the high proportion of *Staphylococcus* isolates in this study that were resistant to ampicillin as compared to the other three antimicrobials. Furthermore, this disparity could be due to differences in the mechanisms of action, with ampicillin targeting the cell-wall while the others targets nucleic acid or protein synthesis [29].

The clustering of antimicrobials from different classes and with different mechanisms of action observed in the preceding paragraph, suggests collateral sensitivity. Collateral sensitivity or cross resistance has previous been reported in other studies [12,13,14,15] and may develop without the target mutations or via mutation in the target resistance protein. Therefore, findings of this study support the evidence of cross resistance or collateral sensitivity among clinical isolates.

Amoxicillin-clavulanic acid and cephalothin also clustered together. This was expected, given that both antimicrobials belong to the β-lactam group and both are highly efficacious towards β-lactamase producing *Staphylococcus* species [31,32]. Likewise, the clustering of clindamycin and lincospectin was anticipated, given that both antimicrobials belong to the same category of antimicrobials called lincosamide. These two antimicrobials are known to be highly efficacious against methicillin-resistant *Staphylococcus aureus* (MRSA) and multidrug resistant staphylococci [32,33].

## 5. Conclusions

In this study it was observed that antimicrobials in the same category or class, share similar efficacy profiles with respect to *Staphylococcus species*. Therefore, it is recommended that when performing susceptibility analysis, laboratories should only include one member of each category or class of antimicrobials to help reduce the cost of antimicrobial susceptibility tests, especially in low resource settings. Likewise, to minimize treatment failures, clinicians are advised not to prescribe antimicrobials belonging to the same group of antimicrobials if one member of that category exhibits reduced efficacy against *Staphylococcus* species.

## Figures and Tables

**Table 1:** Distribution of nonsusceptibility against 15 antimicrobials among 382 *Staphylococcus* species isolated at a veterinary academic hospital in South Africa.

Group	Drug	Frequency	Percent
Aminoglycoside		95	9.2
	Amikacin	28	7.33
	Gentamicin	30	7.85
	Kanamycin	37	9.69
β-lactam			
Penicillins			
	Ampicillin	225	58.9
	Penicillin	212	55.5
Cephalosporine	Cephalothin	29	7.59
Combination	Amoxicillin/clavulanic acid	48	12.57
Tetracycline	Doxycycline	60	15.71
Fluoroquinolones			
	Enrofloxacin	39	10.21
	Orbifloxacin	37	9.69
Potentiated sulfonamide	Co-trimazole^b^	65	17.02
Amphenicol	Chloramphenicol	39	11.34
Lincosamide	Clindamycin	143	37.43
Aminoglycoside-lincosamides	Lincomycin-spectinomycin	170	44.5
Macrolide	Tylosin	40	10.47

**Table 2:** Tetrachronic correlations of the efficacy of 15 antimicrobial agents against *Staphylococcus* isolates at a veterinary academic hospital in South Africa.

Drug	Ami	Amp	Dox	Enr	Gen	Pen	Sul	Chl	Cep	Kan	Cli	Lin	Orb	Syn	Tyl
Ami	1.00														
Amp	0.02	1.00													
Dox	0.35	0.37	1.00												
Enr	0.45	0.16	0.16	1.00											
Gen	0.79	0.37	0.50	0.68	1.00										
Pen	0.02	0.96	0.42	0.09	0.25	1.00									
Sul	0.27	0.50	0.36	0.63	0.69	0.40	1.00								
Chl	0.50	0.37	0.40	0.40	0.48	0.55	0.41	1.00							
Cep	0.60	0.35	0.17	0.25	0.66	0.39	0.25	0.52	1.00						
Kan	0.72	0.26	0.37	0.68	0.93	0.18	0.73	0.50	0.50	1.00					
Cli	0.63	0.38	0.32	0.38	0.60	0.41	0.28	0.60	0.59	0.60	1.00				
Lin	0.61	0.52	0.41	0.43	0.53	0.54	0.31	0.75	0.47	0.64	0.79	1.00			
Orb	0.56	0.30	0.32	0.91	0.70	0.26	0.59	0.46	0.45	0.81	0.60	0.60	1.00		
Syn	0.38	0.63	0.29	0.47	0.69	0.57	0.53	0.54	0.63	0.57	0.38	0.37	0.53	1.00	
Tyl	0.63	0.29	0.41	0.61	0.71	0.34	0.55	0.54	0.72	0.81	0.81	0.71	0.70	0.64	1.00

Ami = Amikacin; Amp = Ampicillin; Dox = Doxycycline; Enr = Enrofloxacin, Gen = Gentamicin, Pen = Penicillin; Sul = Co-trimazole; Chl = Chloramphenicol; Cep = Cephalothin; Kan = Kanamycin; Clin = Clindamycin; Lin = Lincomycin-Spectinomycin; Orb = Orbifloxacin; Syn = Amoxicillin-Clavulanic acid; Tyl = Tylosin.

**Table 3:** Eigenvalues of the correlation matrix.

Eigenvalues of the Correlation Matrix				
	Eigenvalue	Difference	Proportion	Cumulative % of Variance Explained
1	3.479	0.870	0.387	0.387
2	2.609	1.065	0.290	0.676
3	1.543	0.792	0.172	0.848
4	0.752	0.488	0.084	0.931
5	0.263	0.105	0.029	0.961
6	0.159	0.073	0.018	0.978
7	0.086	0.009	0.010	0.988
8	0.077	0.045	0.009	0.997
9	0.032		0.004	1.000

**Table 4:** Loading factors.

Antimicrobial	Factor 1	Factor 2	Factor 3
Enrofloxacin	0.859	−0.146	0.027
Gentamicin	0.898	0.034	0.374
Tylosin	0.801	0.560	0.440
Ampicillin	−0.814	−0.330	0.082
Clindamycin	0.348	0.927	0.189
Lincospectin	0.077	0.848	−0.118
Co-trimazole	0.424	−0.693	0.043
Amoxicillin-clavulanic acid	0.018	−0.360	0.848
Cephalothin	0.248	0.551	0.824

**Table 5:** Results of McDonald’s omega reliability test.

Item	McDonald’s
Ampicillin	0.784
Enrofloxacin	0.771
Gentamicin	0.764
Cephalothin	0.764
Clindamycin	0.741
Lincospectin	0.745
Orbifloxacin	0.762
Amoxicillin-clavulanic acid	0.763
Tylosin	0.749

## Data Availability

The data presented in this study are available on request from the corresponding author. The data are not publicly available because they belong to a third party (the bacteriology lab of the University Pretoria Veterinary Teaching Hospital).
